# The Usefulness of AI-Based Cornea Exposure Rate (CER) Analysis Utilizing the Anigma View System in Evaluating Ptosis Surgery Outcomes

**DOI:** 10.3390/jcm14051691

**Published:** 2025-03-03

**Authors:** JuYoung Park, Hojik Yang, Kyungmin Cho, JungJin Park, Seonghyeon Kim, Myunggyun Seo, Yerim Shin, Gyeonghun Im, Minju Kim, Seung-Han Song, Chang-Wook Seo

**Affiliations:** 1 Department of Plastic and Reconstructive Surgery, Chungnam National University Hospital, Daejeon 35015, Republic of Korea; zoo0park0717@gmail.com; 2 Department of Plastic and Reconstructive Surgery, Chungnam National University Sejong Hospital, Sejong 30099, Republic of Korea; drhjyang@gmail.com; 3 Anigma Technologies Inc., Daejeon 34051, Republic of Korea; ckm@anigma-ai.com (K.C.); jinpark@anigma-ai.com (J.P.); okdalto@anigma-ai.com (S.K.); jonathan@anigma-ai.com (M.S.); shema117@anigma-ai.com (Y.S.); nsoar@anigma-ai.com (G.I.); mjkim@anigma-ai.com (M.K.); 4 Department of Medical Science, College of Medicine, Chungnam National University, Daejeon 35015, Republic of Korea; 5 Department of Plastic and Reconstructive Surgery, College of Medicine, Chungnam National University, Daejeon 35015, Republic of Korea

**Keywords:** ptosis surgery, CER, ImageJ, AI

## Abstract

**Background/Objectives**: Ptosis surgery corrects drooping upper eyelids, improving function and esthetics. Traditional methods like marginal reflex distance (MRD) and palpebral fissure height (PFH) offer limited one-dimensional measurements. This study evaluates AI-based corneal exposure ratio (CER) analysis, a two-dimensional approach, compared to manual ImageJ methods for assessing ptosis surgery outcomes. **Methods**: In this prospective study, 100 eyes from 50 patients were analyzed using both methods. AI-based CER measurements were compared to manual ImageJ measurements for reliability and accuracy. **Results**: AI-based CER measurements were comparable to manual ImageJ, with high reliability (ICC 0.992, 0.985). Preoperative CER was 55.34% (manual) and 55.79% (AI), increasing to 75.92% (manual) and 75.84% (AI) postoperatively. The AI tool showed minimal bias and high repeatability (ICC 1.000), offering faster automated measurements. **Conclusions**: AI-based CER analysis matched manual methods in accuracy but provided significant efficiency advantages, making it suitable for clinical use. Limitations include a small homogeneous sample size and reliance on 2D imaging, which may not fully capture three-dimensional changes. Further studies are recommended to enhance generalizability and precision.

## 1. Introduction

Among Asians, ptosis surgery is one of the most performed plastic surgery procedures. This surgery corrects drooping of the upper eyelid, improving both function and appearance. As the frequency of ptosis surgeries increases, surgeons continually strive to minimize complications and enhance patient satisfaction, with accurate measurements playing a crucial role in achieving these goals.

Retrospective analyses of before and after photos are typically employed to evaluate the outcomes of ptosis surgery. These analyses are critical for evaluating the effectiveness of the procedure, determining patient satisfaction, and guiding further surgical improvements.

Common approaches for assessing ptosis surgery outcomes typically involve metrics such as the marginal reflex distance (MRD) and palpebral fissure height (PFH) [1,2]. MRD1, which quantifies the distance from the pupil’s center to the upper eyelid margin, is a widely recognized metric for assessing upper eyelid positioning. Likewise, PFH measures the vertical distance between the upper and lower eyelid margins at the pupil’s center. However, both of these metrics are limited to one dimension and may not adequately reflect the intricate three-dimensional changes in the eye’s appearance following surgery.

**Figure 1:**
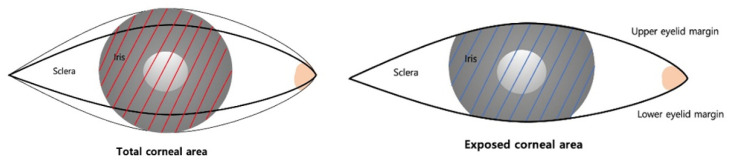
Schematic drawing of eyelid parameters. CER: corneal exposure ratio, the proportion of the exposed corneal area relative to the total corneal area. The red line represents the Total Corneal Area, and the blue line represents the Exposed Corneal Area.

The corneal exposure ratio (CER) provides a more comprehensive two-dimensional measurement. It represents the proportion of the exposed corneal area relative to the total corneal area, offering a clearer reflection of the overall appearance and changes in corneal exposure in two dimensions (Figure 1).

The CER may also serve as an esthetic measure. Previous research from the 1960s found that faces with larger corneal exposure were perceived as more appealing and attractive [3]. This suggests that the CER could be an important factor not only in functional outcomes but also in assessing the esthetic success of ptosis surgery.

Conventionally, software like ImageJ has been used for measuring eye-related metrics in pre- and post-surgical photos. ImageJ allows for high accuracy through manual measurements, making it a reliable tool in research [4]. However, the reliability of ImageJ measurements can vary depending on the user’s expertise, and the process is labor-intensive and time-consuming, especially when dealing with large datasets. These factors limit its utility in busy clinical settings, making ImageJ less practical for real-time clinical applications.

Recent advancements in artificial intelligence (AI) have offered promising solutions to the limitations of conventional tools like ImageJ [5]. AI-based tools for image analysis can automate the measurement process, providing faster and more consistent results for measurements in pre- and post-surgical images. AI tools offer significant advantages, including speed and simplicity. Measurements that previously took minutes or hours can now be obtained almost instantaneously. This efficiency is particularly valuable in clinical environments where quick decision-making is essential. Additionally, AI tools reduce human error by relying on algorithms to identify features such as the corneal area or eyelid margin, ensuring greater consistency and accuracy. AI tools can also handle large datasets, making them scalable for studies involving many patients or images. As AI technology advances, it is likely to become integral to ptosis surgery analysis, enhancing workflow and precision in outcome assessments.

In this study, we propose to analyze CER before and after ptosis surgery using AI-based tools. AI tools offer a faster and more automated alternative to the conventional ImageJ method, making them highly suitable for both clinical applications and research involving large datasets. With their ability to quickly and accurately measure CER, AI tools have the potential to streamline outcome assessments, improving both efficiency and accuracy in surgical evaluations.

## 2. Methods

### 2.1. Patients

This study involved 100 eyes from 50 patients aged over 19 years who visited Chungnam National University Hospital between 2023 and 2024. The study followed the ethical guidelines of the Declaration of Helsinki. Informed consent was obtained from all participants, including consent for potential publication in both online and print formats. The study was approved by the Ethics Committee of Chungnam National University Hospital, Daejeon, Republic of Korea (CNUH-IRB no. 2023-06-098). Patient demographic information (Table 1), such as age, sex, and any history of plastic surgery, was collected and analyzed.

This prospective observational study included 50 patients (25 men and 25 women) aged between 19 and 80 years, all diagnosed with bilateral ptosis. A total of 100 eyes were evaluated in the study. Among the patients, 33 had equal ptosis severity in both eyes, while 17 exhibited varying degrees of severity between their eyes.

The severity of ptosis was categorized based on the degree of upper eyelid drooping. Mild severity was defined as drooping of 1–2 mm, while moderate-to-severe ptosis was classified as drooping greater than 3 mm. In total, 51 eyes were categorized as having mild ptosis, and 49 eyes were classified as moderate to severe [6].

This study retrospectively analyzed preoperative photographs and photographs taken six months after surgery. All surgeries were performed by a single expert with over 20 years of experience. Patients with a history of previous eye surgeries or facial trauma were excluded from the study.

### 2.2. Image Acquisition

Frontal view digital photographs, both pre- and post-operative, were captured for all patients using a digital camera with a standardized lens (Canon 1500 D, Canon Corporation, Tokyo, Japan, 24–120 mm) by the same photographer. During the photo sessions in our studio, patients were seated 1.0 m from the camera, maintaining a natural head position (NHP) and aligned along the same horizontal axis. All images were taken with the patients’ faces at rest and their frontalis muscles fully relaxed. Photographs were collected at two specific time points, namely before surgery and on postoperative day 180.

### 2.3. Manual Measurements

**Figure 2:**
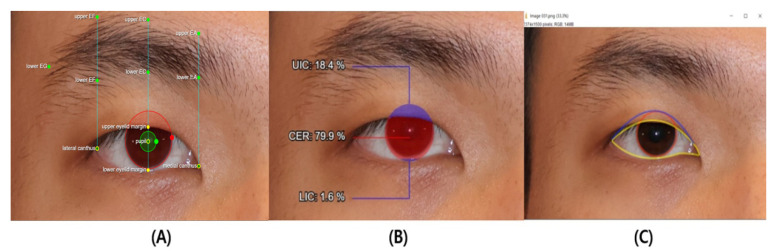
Eyelid measurements using the AI tool and manual tool. (**A**) Eyelid parameters generated by the AI tool (Anigma-view version 1.0.6). (**B**) CER measurement using the AI tool (Anigma-view version 1.0.6). (**C**) CER measurement using the manual tool (ImageJ version 1.46).

The preoperative and postoperative images were processed and analyzed using Java-based image analysis software ImageJ version 1.46 (National Institutes of Health, Bethesda, MD, USA). The CER (corneal exposure ratio) for both preoperative and postoperative images was manually measured by the same expert using ImageJ (Figure 2).

The software was used to convert pixel measurements into millimeters, using the interlimbal distance of the cornea, set at 11.7 mm [7], as the reference point in each photograph.

### 2.4. AI Tool Measurements 

**Figure 3:**
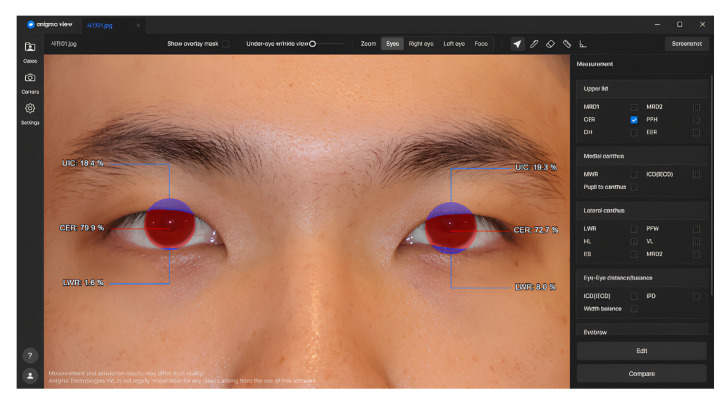
Analysis screen of Anigma-view version 1.0.6.

In this study, we utilized Anigma-view version 1.0.6 (Anigma Technologies Inc., Daejeon, Republic of Korea), an AI-based tool designed for facial image analysis, to measure the corneal exposure ratio (CER) before and after ptosis surgery (Figure 2 and Figure 3) [8]. The next section outlines the key components and procedures involved in the measurement process.

We utilized a DeepLabV3+ model [9] with fine-tuning to perform semantic segmentation of the eye regions. The model was trained on a dataset comprising images from patients who had provided consent for the use of their data. The training process spanned 10,000 iterations, employing the Adam optimizer with a learning rate set at 0.0004.

#### 2.4.1. Image Alignment and Segmentation

**Figure 4:**
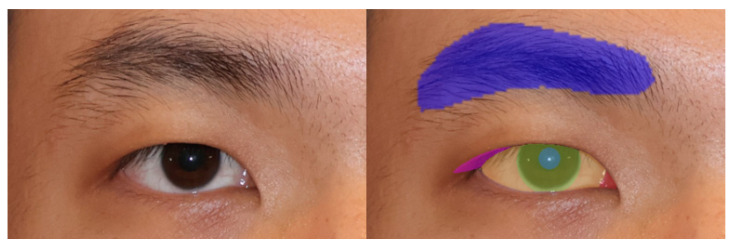
The cropped eye and segmented eye image example by Anigma-view version 1.0.6.

All input face images were first processed through a face alignment function within the Anigma-view program, ensuring that each image was standardized in terms of position and angle, allowing for consistent measurements across samples. Following the approach of Lou et al. [10], we used the Face Alignment [11] open-source project to localize the eye regions in the input images. To specifically focus on the eye features, we cropped the eye area to a 512 × 512 resolution, ensuring that our analysis was limited to the relevant regions. The localized eye regions were then processed by our fine-tuned DeepLabV3+ model to generate segmentation masks for the eyelid and cornea, providing a detailed delineation of eye structures necessary for accurate CER calculations. See Figure 4 for an example of the segmented eye image. Once aligned and segmented, each image underwent further analysis for CER calculation using advanced segmentation techniques in Anigma-view.

#### 2.4.2. Circle Detection and Masking

To accurately measure the pupil and iris, we applied a well-established circle detection algorithm [12]. This method identifies the center coordinates (x, y) and radius of circular objects, such as the pupil and iris, through the following steps:

1. Edge detection: we used an edge detection algorithm [13] to highlight the edges in the segmented image.
2. Voting in Hough space: For each detected edge pixel (x, y), the algorithm calculates possible circles by voting in the Hough parameter space. This process involves varying the radius (r) and identifying the center points (a, b) using the following equations: a = x – rcos (θ), b = y – rsin (θ), where θ ranges from 0 to 360°.
3. Circle identification: peaks in the Hough parameter space are detected, corresponding to the most likely circle centers and radii.
4. Return values: the algorithm returns the coordinates (x, y) and radius of the detected circles.

#### 2.4.3. Corneal Exposure Ratio (CER) Calculation

To compute the corneal exposure ratio (CER), we first detected the pupil and iris using the circle detection method. We then created a cornea mask by combining the detected pupil and iris with their respective masks. The white area of the eye was also segmented to further refine the exposed eye mask.

Using a Boolean mask, we initialized the full iris mask by setting each pixel within the iris radius to True. The cornea mask was then updated by performing a logical AND operation between the full iris mask and the exposed eye mask.

The final step involved calculating the CER as the ratio of the cornea area to the full iris area. This was achieved by counting the non-zero elements in the respective masks and multiplying the result by 100 to obtain the percentage of exposed cornea.

In conclusion, this method allowed for a precise and reliable measurement of the corneal exposure ratio (CER) before and after ptosis surgery, providing critical insights into the surgical outcomes.

### 2.5. Statistical Analyses

The mean values of CER measurements were calculated, and the differences between pre- and post-surgery CER values were analyzed using a two-paired *t*-test. Statistical significance was defined as *p* < 0.05.

The reliability between the AI tool and manual measurements of CER was analyzed using a one-way ANOVA test. Intraclass correlation coefficients (ICCs) were used to quantify the agreement between CER measurements obtained from ImageJ and the AI tool, as well as between the AI tool’s first and second CER measurements. The ICC values were interpreted as follows: values between 0.41 and 0.6 indicated moderate agreement, values between 0.6 and 0.8 indicated substantial agreement, and values between 0.8 and 1.0 indicated excellent agreement. Bland–Altman plots were generated to visually compare the differences between CER measurements obtained from ImageJ and the AI tool, as well as between the first and second CER measurements performed by the AI tool.

The improvement rate of CER before and after surgery based on ptosis severity was analyzed using a two-way ANOVA test.

All data were analyzed using the Statistical Package for the Social Sciences (SPSS) version 26 (IBM Corporation, Armonk, NY, USA) and Microsoft Excel version 16.84 (Microsoft^®^, Redmond, WA, USA).

## 3. Results

### 3.1. Pre- and Post-Operative CER Comparison

The corneal exposure rate (CER) values measured manually using ImageJ and repeated measurements using an AI-based tool before and after ptosis surgery can be seen in Table 2. The preoperative manual measurement of the CER was 55.34 ± 111.71%, while the postoperative manual measurement increased to 75.92 ± 7.55%. Similarly, the preoperative CER values obtained from two repeated measurements using the AI tool were both 55.79 ± 11.51%, and the postoperative AI tool measurements were 75.84 ± 7.40% for both repetitions.

The CER values measured by AI-CER 1st, AI-CER 2nd, and ImageJ showed significant differences before and after surgery, as determined by the two-paired *t*-test (*p* < 0.001).

### 3.2. Reliability Between Two Measurements of CER

The CER values measured using manual and AI-based methods showed close agreement. The mean values obtained from the three methods (manual, AI-CER 1st, AI-CER 2nd) exhibit statistically insignificant differences, as determined by a one-way ANOVA test (*p* > 0.5) (Table 3).

The intraclass correlation coefficients (ICCs) between the manual and AI-based tool measurements ranged from 0.988 to 0.995 (preoperative) and 0.978 to 0.990 (postoperative), indicating excellent reliability between the two methods (Table 4). Moreover, the ICCs between the two repeated AI-based tool measurements reached 1.000, demonstrating the AI-based tool’s high repeatability.

**Figure 5:**
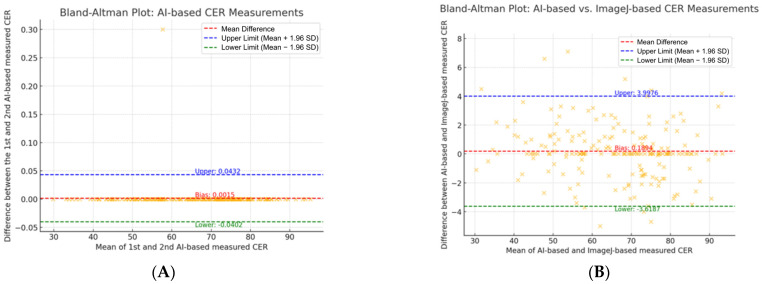
Bland–Altman analysis between different CER measurements. (**A**) Bland–Altman plot between AI tool 1st measurement and 2nd measurement. (**B**) Bland–Altman plot between AI tool and ImageJ measurement.

The Bland–Altman plot also confirmed the consistency between two measurements (Figure 5). The bias [95% limits of agreement (LoAs)] between the manual and AI-based tool measurement was −3.619 and 3.998, and the bias (95% LoAs) range between repeated AI-based tool measurements was −0.040 and 0.0043. These results highlight both the consistency between the measurement methods and the exceptional reproducibility of the AI tool.

### 3.3. Improvement Rate of CER Before and After Surgery Based on Ptosis Severity

**Figure 6:**
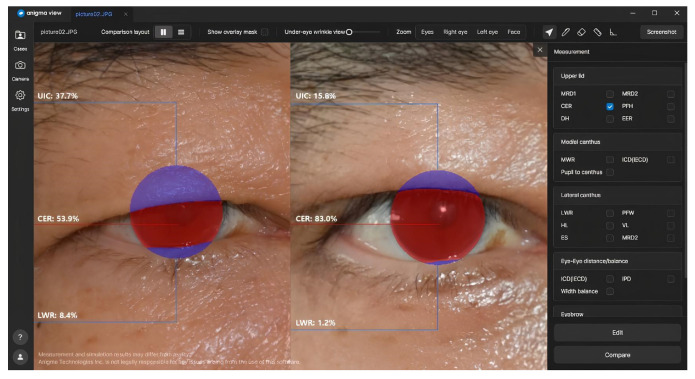
Comparison of preoperative and postoperative CER using Anigma-view version 1.0.6.

**Figure 7:**
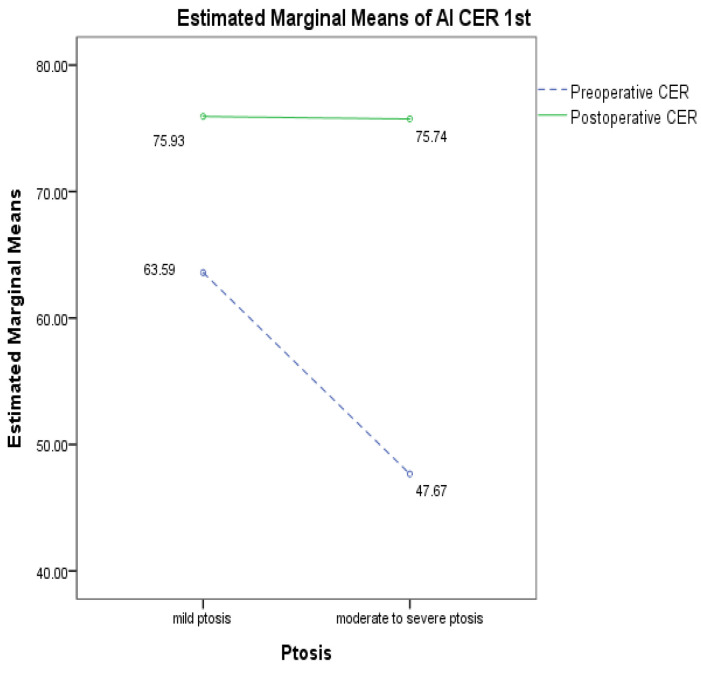
Comparison of preoperative and postoperative CER changes based on ptosis severity using AI-based tool 1st.

This study analyzed 100 eyes from 50 patients, categorizing them as having mild (51 eyes) or moderate-to-severe (49 eyes) ptosis. Table 5 represents the changes in CER values pre- and post-surgery based on the severity of ptosis by using AI tool measurement (Figure 6). For patients with mild ptosis, the CER increased by 19.41%, from 63.59 ± 8.64% preoperatively to 75.93 ± 7.49% postoperatively. For patients with moderate-to-severe ptosis, the CER increased by 58.88%, from 47.67 ± 7.97% to 75.74 ± 7.38 (Table 5). These findings suggest that the improvement in CER after surgery was greater in patients with moderate-to-severe ptosis compared to those with mild ptosis (Figure 7).

## 4. Discussion

In plastic surgery, accurate pre- and post-operative comparison and evaluation are essential for measuring surgical outcomes and ensuring patient satisfaction. This study emphasizes the need of using AI-based tools for assessing the cornea exposure rate (CER) and evaluates their precision and efficacy in comparison to conventional methods like ImageJ-based analysis.

In prior research, several studies have used ImageJ to measure the CER. Kim et al. (2016) [14] assessed the CER as an important metric for evaluating outcomes following frontalis muscle transfer. Ki Soo Park et al. (2020) studied the CER in relation to age and gender in Korean populations [15], providing insights into how these factors influence the CER.

Additionally, there has been growing interest in research utilizing AI-based methods. Ji Shao et al. (2024) [16] used image-based deep learning to analyze MRD1 and MPLD (mid-pupil lid distances). Nam et al. (2024) [17] employed neural network-based automatic tools to analyze MRD1, MRD2, upper eyelid length, and lower eyelid length. Lixia Lou et al. (2021) [10] applied deep learning-based AI tools to analyze MRD1, MRD2, and upper eyelid length. However, to date, no studies have utilized AI tools specifically to analyze the CER.

This study is essential, as it compares conventional ImageJ-based methods with AI-based tools for assessing the CER before and after ptosis surgery. In conclusion, our results demonstrate that CER measurements obtained using AI-based methods did not significantly differ from those obtained using ImageJ. This shows that AI tools could provide comparable outcomes to conventional methods of evaluation.

This indicates that AI-based methods are as accurate as conventional methods while also providing the benefits of speed and simplicity for clinical applications. The ability of AI tools to simultaneously analyze large volumes of images makes them particularly useful in research as well.

Furthermore, the potential application of AI tools extends beyond ptosis surgery. It can be applied not only in CER but also across a wider range of eye measurement metrics in various types of plastic surgeries.

The incorporation of AI-based tools in surgical assessment can not only enhance the objectivity of surgical outcomes for plastic surgeons but also provide patients with clear, objective metrics, potentially increasing their satisfaction. Additionally, AI-driven image analysis can be widely applied in both cosmetic and reconstructive plastic surgery.

However, our study has limitations. First, the study included only 50 patients and 100 eyes, all of whom were Korean and from a single institution, which limits the generalizability of the findings. Future studies should involve larger, more diverse populations across multiple institutions. Second, variations in patient posture and distance from the camera during photo capture may have introduced errors. Standardizing photo-taking procedures to ensure consistent posture, distance, and camera settings is essential for more accurate measurements. Third, relying on 2D photo analysis may have caused some distortion in capturing the 3D structure of the eyes. Future research should explore 3D imaging technologies to provide a more comprehensive analysis.

## 5. Conclusions

This work proposes a novel approach to analyze the effects of ptosis surgery with AI tools for CEA measurement. The AI system demonstrated speed, objectivity, and complete automation, making it an effective tool for comparing preoperative and postoperative outcomes. It is essential in clinical practice to be able to convey surgical outcomes to patients in a timely and understandable manner, and AI-based CEA analysis will help to achieve this goal. Additional research with larger, more diverse populations and advanced 3D imaging is necessary to enhance the accuracy and applicability of this method, even though our findings support the use of AI tools in this context.

## Figures and Tables

**Table 1:** Patient demographics and severity.

Dermographics	
No. of patients (no. of eyelids)	50 (100)
Age (yr)	46.9 ± 18.7
Sex (male/female)	25/25
Severity of ptosis (no. of eyelids)	
Mild (≤2 mm)	51
Moderate to severe (>2 mm)	49

Values are presented as mean ± standard deviation.

**Table 2:** The comparison in pre- and post-surgery results for AI-CER 1st, AI-CER 2nd, and ImageJ CER.

	Preoperative	Postoperative	t
AI-CER 1st	55.79 ± 11.51	75.84 ± 7.40	−19.477 ***
AI-CER 2nd	55.79 ± 11.51	75.84 ± 7.40	−19.477 ***
ImageJ-CER	55.34 ± 11.71	75.92 ± 7.55	−19.173 ***

The statistical significances were evaluated by the two-paired *t*-test, expressed as mean ± standard deviation. ***: *p* < 0.001.

**Table 3:** The comparison of CER values based on the measurement method.

	M ± SD	F
AI-CER 1st	65.81 ± 13.94	0.011
AI-CER 2nd	65.81 ± 13.94	
ImageJ CER	65.63 ± 14.25	

Statistical significances were evaluated by one-way ANOVA, mean ± standard deviation.

**Table 4:** Intraclass correlation coefficients between two measurements of CER preoperatively and postoperatively.

	ICC	(95% CI)
pre		
1st and 2nd	1.000	(1.000–1.000) ***
1st and ImageJ	0.992	(0.988–0.995) ***
post		
1st and 2nd	1.000	-
1st and ImageJ	0.985	(0.978–0.990) ***

***: *p* < 0.001; -: the data of the two variables are in agreement.

**Table 5:** Comparison improvement rate of CER before and after surgery using AI-based tool 1st.

	Preoperative CER	Postoperative CER	F
Total eyes of ptosis	55.79 ± 11.51	75.84 ± 7.40	52.163 ***
Mild ptosis	63.59 ± 8.64	75.93 ± 7.49	
Moderate-to-severe ptosis	47.67 ± 7.97	75.74 ± 7.38	

(Mild = 51, moderate-to-severe ptosis = 49). Statistical significances were evaluated by two-way ANOVA, mean ± standard deviation. ***: *p* < 0.001.

## Data Availability

The data presented in this study are available upon request from the corresponding author. The data are not publicly available due to patient’s privacy.

## Abbreviations

The following abbreviations are used in this manuscript:

AI	Artificial Intelligence
MRD	Marginal Reflex Distance
PFH	Palpebral Fissure Height
CER	Corneal Exposure Ratio
NHP	Natural Head Position
ICC	Intraclass Correlation Coefficient
